# REWRITALIZE your recovery: a study protocol for a randomised controlled trial (RCT) examining the effectiveness of the new recovery-oriented creative writing group intervention REWRITALIZE for people with severe mental illness

**DOI:** 10.1186/s12888-024-06254-5

**Published:** 2024-12-05

**Authors:** Susanne Henningsson, Jon Toke Brestisson, Siv-Therese Bogevik Bjørkedal, Birgit Bundesen, Keld Stehr Nielsen, Bea Ebersbach, Carsten Hjorthøj, Lene Falgaard Eplov

**Affiliations:** 1 Danish National Centre for Arts and Mental Health, Mental Health Center Amager, Hans Bogbinders Allé 3, 2.tv, 2300 Copenhagen S, Denmark; 2 Copenhagen Research Unit for Recovery, Mental Health Center Amager, Hans Bogbinders Allé 3, 3, 2300 Copenhagen S, Denmark; 3 Copenhagen Research Center for Mental Health, Mental Health Center Copenhagen, Gentofte Hospitalsvej 15, 2900 Hellerup, Denmark; 4 Department of Public Health, Section of Epidemiology, University of Copenhagen, Øster Farimagsgade 5, 1353 Copenhagen K, Denmark

**Keywords:** Participatory arts, Creative writing, Recovery, Psychosocial interventions, Severe mental illness, Schizophrenia spectrum disorders**.**

## Abstract

**Background:**

Health institutions advocate for psychosocial and recovery-oriented interventions alongside pharmacological treatment for severe mental illness. Participatory arts interventions appear promising in promoting personal recovery by fostering connectedness, hope, renegotiation of identity, meaning-making, and empowerment. Despite encouraging findings, however, the evidence base remains thin. Further, results from cognitive literature studies suggest possible impact on parameters of clinical recovery, but this has not been studied in clinical contexts. We developed REWRITALIZE, a structured, recovery-oriented, fifteen-session creative writing group intervention led by a professional author alongside a mental health professional. Participants engage with literary forms, write on them, share their texts, and partake in reflective discussions within a supportive, non-stigmatising environment, designed to promote self-expression, playful experimentation, agency, recognition, participatory meaning-making, renegotiation of identity and social engagement.

The aim of this project is to evaluate REWRITALIZE for persons with severe mental illness through a randomised controlled trial (RCT) focusing on personal recovery outcomes. Additionally, an embedded pilot RCT will explore additional outcomes i.e., clinical recovery for a subgroup with schizophrenia spectrum disorders.

**Methods:**

The RCT is an investigator-initiated, randomised, two-arm, assessor-blinded, multi-center, waiting-list superiority trial involving 300 participants (age > 18) from six psychiatric centers in regions Capital and Zealand in Denmark, randomised to receive either the creative writing intervention combined with standard treatment or standard treatment alone. Assessments will be conducted before and after the intervention and at six months post intervention. The primary outcome is personal recovery at the end of intervention measured with the questionnaire of the process of recovery. Secondary outcomes include other measures of personal recovery, self-efficacy, mentalising, and quality of life. The pilot RCT, integrated within the RCT, will focus on 70 of the participants aged 18–35 with schizophrenia spectrum disorders, evaluating exploratory measures related to perspective-taking, social cognition, cognitive function, psychosocial functioning, and symptom pressure.

**Discussion:**

This is the first RCT for creative writing groups. It assesses whether REWRITALIZE, as adjunct to standard mental healthcare, is more effective for personal recovery than standard care. If successful, it would provide evidence for the efficacy of REWRITALIZE, potentially enabling its implementation across mental health centers in Denmark.

**Trial registration:**

Privacy (data protection agency): p-2023–14655.

Danish National Center for Ethics: 2313949.

Clinicaltrials.gov: NCT06251908. Registration date 02.02.2024.

## Background

Guidelines for aiding individuals with mental disorders stress the need to supplement pharmacological treatment with psychosocial interventions and recovery-oriented approaches [71–73, 106]. The implementation of recovery-oriented practices in mental healthcare is, however, still in an early phase.

Recovery comprises two main categories – clinical recovery and personal recovery. Clinical recovery refers to a reduction of symptoms of mental disorders and a restoration of functioning, including cognitive, social, and occupational functioning. Personal recovery refers to the process of constructing a personally meaningful life within and beyond the limits of one’s mental disorder [2, 5, 26, 103]. Five key aspects of personal recovery have been identified and summarised in the CHIME framework: development and maintenance of supportive relationships that facilitate the experience of belonging (Connectedness), motivation and belief in one’s ability to achieve change (Hope), building a positive self-conception and overcoming stigma (Identity), living a meaningful life with fulfilling activities (Meaning), and developing autonomy and taking responsibility and control over one’s life (Empowerment) [54]. People with severe mental illness understand recovery as a transformation from a negative self-conception marked by helplessness to a more positive self-conception of wellbeing and emphasise the importance of both clinical recovery and the CHIME processes of personal recovery for this transformation [27]⁠.

Recovery-oriented interventions that consider social difficulties for persons with severe mental illness are still not systematically implemented in mainstream psychiatry [25, 56, 85]. A recent review could only identify few recovery-oriented services [5]. Amongst them were art-based interventions [72]⁠. Studies of different kinds of art therapy have shown mixed results (e.g. [22, 64])⁠. Our focus is on participatory arts and how it can be used in mental healthcare. In participatory arts, the artistic activity is facilitated by artists in a group setting, the goal of the activity is understood as engaging in the artistic process, and the participants share and engage in reflective discussion about the produced art works ([96, 14, 77, 105]). A review of qualitative studies on recovery outcomes for participatory arts activities suggests that participatory arts might be beneficial for the CHIME processes [105]. A review of qualitative, quantitative, and mixed methods studies on art-based practices, including participatory arts, support positive effects on the CHIME processes Identity and Connectedness [110]⁠. The evidence-base from quantitative studies on participatory arts is, however, thin: No randomised controlled trial has been conducted [36, 76]. Furthermore, regarding domains relating to clinical recovery, e.g. social and cognitive functioning, research is also lacking. Social and cognitive difficulties are especially challenging and pertinent for persons suffering from schizophrenia spectrum disorders, and interventions targeting these issues are recommended for this population, but available interventions and evidence for their effectiveness are scarce [4, 20, 31, 34, 62, 72]. Cognitive literature studies, investigating the impact of engaging with literature on cognition, suggest that reading literature may further social cognitive capacities [32, 33, 49, 50] and that writing literature is associated with higher levels of mentalising capacities [61, 68]. This has, however, neither been researched within a clinical population nor a controlled setting. Hence the evidence from empirical cognitive literature studies may be promising.

When it comes to creative writing interventions in healthcare contexts, writing in groups has been suggested to contribute to recovery [37, 51, 92]⁠⁠. A review on quantitative and qualitative findings regarding creative writing groups and recovery, indicated beneficial effects on the CHIME factors connectedness, empowerment, and identity [70]⁠. The one study that considered participatory arts, i.e., creative writing groups led by a professional author, reported changes in wellbeing from pre to post intervention [113]⁠. A metareview conducted by the British NHS, including studies on both somatic and mental disorders, suggested that writing that is not facilitated by a trained leader might not promote health, but that facilitated writing requires more exploration [76]. In line with this, further supporting the participatory arts format, others have underlined the importance of using professional authors as facilitators [51]. In conclusion, randomised controlled studies are needed to assess if there are beneficial effects of creative writing groups on recovery [18, 45, 70].

The current intervention, REWRITALIZE, is a creative writing group intervention that is conducted by a professional author and focuses on the text as aesthetic form (see Intervention). Participants are instructed to respect the narrative distance, i.e., the distance between the narrated self and the self who narrates [38]⁠ and discuss the text rather than the person who wrote it. The protective distance between text and personal experiences is meant to establish a safe space in which participants can engage with artistic work on their own terms and express themselves spontaneously in both writing and reflective discussions about others’ art works. This is expected to allow participants to put into words difficult feelings or experiences, to practice mentalisation in reflective sessions, i.e., to reflect on their own and others’ mental states, relate to a plurality of perspectives and integrate others’ perspective into their own outlook [28, 46, 110], and to promote their self-efficacy ⁠[8, 9, 112]. Together this may reduce self-stigmatisation and illness identity, stimulate participatory meaning-making, enhance connectedness between the participants and inspire renegotiation of identity into a more positive self-conception, thus contributing to personal recovery [30, 43, 55, 57–59, 65, 67, 81, 91, 94, 98, 110, 113, 118, 119, 117]. Following studies in non-clinical populations pointing to reading and writing literature having possible impacts on social cognitive factors, it will be valuable to explore if the characteristics of the REWRITALIZE creative writing group intervention may also contribute to clinical recovery, i.e. cognitive and psychosocial functioning and symptom reduction.

**Fig. 1:**
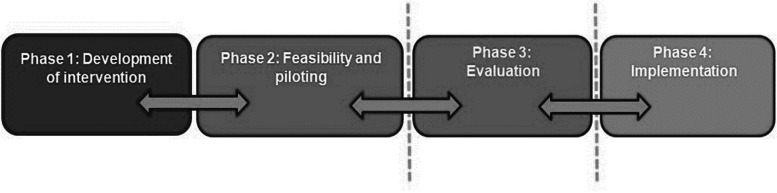
The design of the research program

The present study is part of a larger research program that has developed recovery-oriented, manualised, participatory arts groups, including REWRITALIZE with creative writing as artistic format [16]. The four phased design of this research program (Fig. 1) is based on recommendations from an international group of leading artist, arts researchers, and healthcare researchers, promoting complex interventions in healthcare treatment [36, 101, 102].

Phases 1 and 2 have been carried out. This protocol is a description of the research to be carried out in phase 3. The overarching aim of the present study is to evaluate the newly developed recovery-oriented, manualised, participatory writing group-format REWRITALIZE for persons with severe mental illness. The evaluation of effectiveness will be carried out as a randomised clinical controlled trial (RCT).

### Objectives

- Objective 1 of this study is to determine if REWRITALIZE as supplement to standard mental healthcare is more effective than standard mental healthcare alone for promoting personal recovery, as measured by the Questionnaire for the process of recovery (QPR) immediately after the end of the intervention, in a sample of individuals with severe mental illness.
- Objective 2 is to investigate if REWRITALIZE as supplement to standard mental healthcare is more effective than standard mental healthcare alone in promoting mentalising, self-efficacy, functioning and quality of life, as measured at the end of the intervention, and in promoting personal recovery six months after the end of the intervention.
- Objective 3 is to explore REWRITALIZE as supplement to standard mental healthcare for advancing clinical recovery variables, specifically cognition and social cognition, and pilot-test if REWRITALIZE leads to symptom reduction and improve psychosocial function in a smaller sample of participants with schizophrenia spectrum disorders.

## Methods

The study is designed as an RCT with an embedded pilot RCT. Objective 1 and 2 will be investigated in the RCT, while objective 3 will be investigated in the pilot RCT. Below follows detailed description of the RCT and the pilot RCT.

### RCT design

**Fig. 2:**
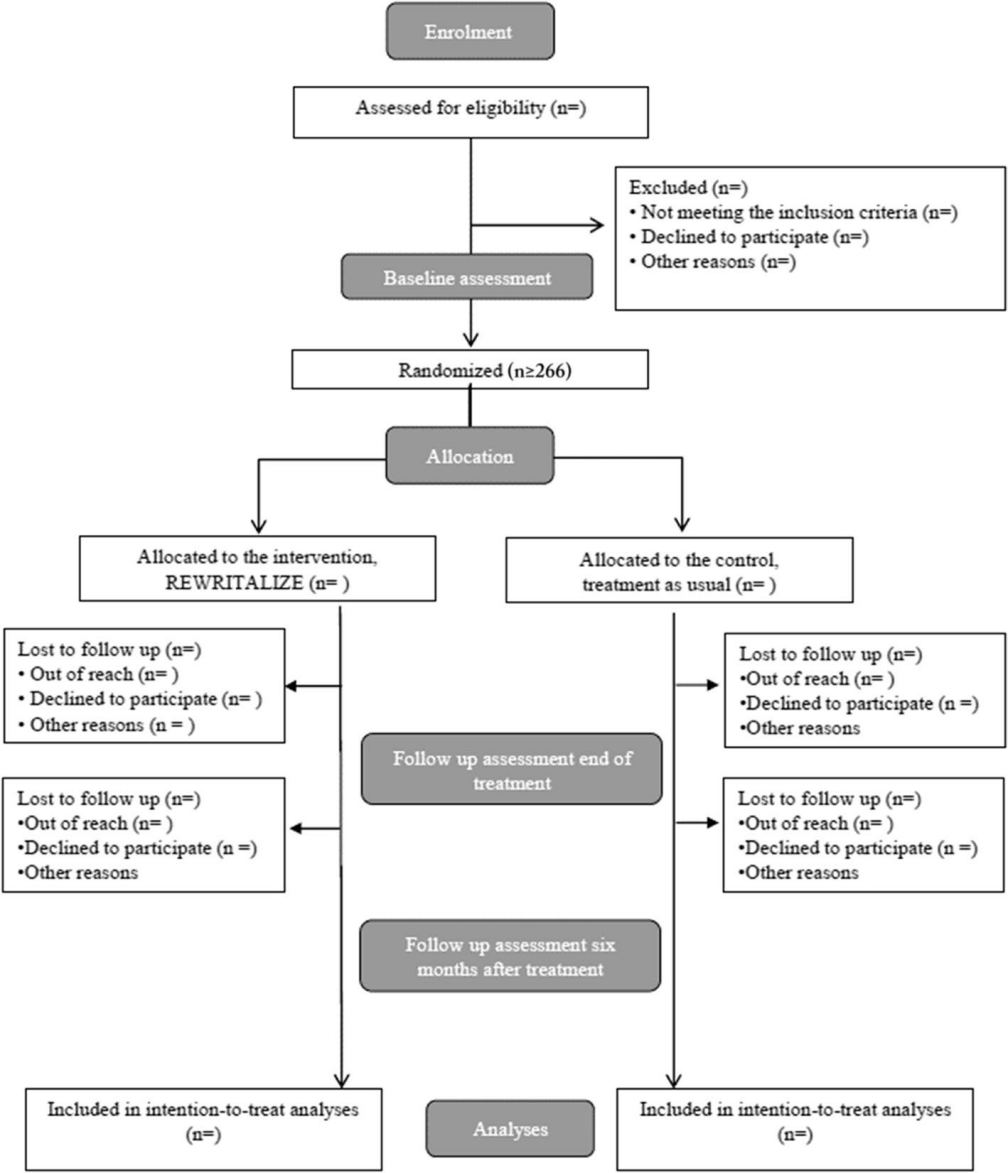
Trial flowchart

The RCT is designed as an investigator-initiated, randomised, two-arm, assessor-blinded, multi-center, waiting list-controlled superiority trial. The allocation ratio to active and control condition is 1:1. The participants (*n* = 300) will be randomised to active condition (REWRITALIZE plus standard treatment) or waiting list control condition (standard treatment) (see Randomisation below). As it is a waiting list control group, the persons randomised to the control group will be invited to participate in a creative writing group after the last assessment (within one year). To ensure high methodological quality, the trial is designed and will be reported according to the SPIRIT 2013 Statement and the modified CONSORT 2017 criteria for non-pharmacological trials [15, 17]. Data will be collected at baseline, at the end of the intervention (approximately 4.5 months after baseline) and six months after the end of the intervention (approximately 10.5 months after baseline). A flow chart of the study is provided in Fig. 2.

### Pilot RCT design

The pilot RCT investigates whether the REWRITALIZE intervention may further social cognition, cognition, psychosocial functioning, and symptom reduction in a subsample of participants with schizophrenia spectrum disorders (*n* = 70). Given the novelty of this area, key uncertainties must be clarified [35] concerning feasibility of the chosen measures, data collection, and analysis. The pilot RCT thus investigates whether the measures on cognition and social cognition are feasible in this setting. The pilot RCT also measures key domain outcomes, i.e. mean, variation, between group differences at end of intervention and within group differences pre and post intervention. The pilot RCT will, furthermore, ascertain whether data will be normally distributed in this population using these measures, and whether ceiling effects may emerge. As the pilot RCT is embedded within the RCT it will follow the overall procedures regarding recruitment, randomisation, and data collection. Data will be collected at baseline and post-intervention follow-up.

### Participants

Participants in the RCT will be included in the study if they are over 18 and diagnosed with a severe mental illness, i.e., schizophrenia, schizotypal, delusional, and other non-mood psychotic disorders, bipolar disorder, severe depression, posttraumatic stress disorder, or borderline personality disorder (F2, F31, F32.2, F32.3, F33.2, F33.3, F43.1, F60.3). Forensic psychiatric patients will be excluded. In all, 30–34 groups, 15–17 active groups and 15–17 control groups, will be carried out with 8–10 participants in each group. Although power calculations (see below: Sample size and power calculations) set the minimum sample size to 266 and the RCT intention-to-treat principle reduces the threat of drop-out for power, we will aim to include 300 participants. Considering the relatively high drop-out rate in similar interventions [64, 110], the enhanced inclusion enables us to aim for a higher number of real, as opposed to imputed, follow-up data, and to minimise the risk to end up with low power if participants were to retract their informed consent.

Participants in the pilot RCT will be included in the study if they are between 18 and 35 and fulfil the criteria for a schizophrenia spectrum disorder (F20, F21) according to the Present State Examination [87, 115].

Participants will be recruited from outpatient clinics’ OPUS programs (early intervention program for first episode psychosis) and F-ACT (Flexible Assertive Community Treatment) at Mental Health Centres in Regions Capital and Zealand in Denmark [40, 75, 109].

### Intervention

REWRITALIZE is conceptualised as a hybrid between an art group and a group therapy intervention [16]. It is developed by an interdisciplinary group of professional authors, user experts, and researchers, and it is manualised (Bundesen B, Llambías P, Rosenbaum B: Manual for REWRITALIZE, forthcoming).

REWRITALIZE consists of 15 three-hour sessions, 14 weekly with an additional session after a one-month break to reduce the risk of adverse effects at the end of the intervention. During the sessions, the 8–10 participants are presented with literary forms that function as prompts for writing exercises. Participants write spontaneously on the presented form for 5–15 min. After each text is read out loud, the participants engage in a reflective discussion about the text. The primary conductor, a professional writer with teaching experience, secures high artistic standards. S/he is supplemented by a co-conductor with clinical expertise who participates in the group activities together with the other participants and ensures psychological safety. Together they constitute the conductor-tandem. The exercises are presented during the course of sessions to participants in an order securing adherence to group therapeutic principles.

The design of the intervention aims at providing a safe, non-judgmental, non-stigmatising space in which illness is not in focus, a space that demotes performance anxiety and promotes playfulness, spontaneous expression, openness to otherness and a plurality of perspectives. This is achieved by several characteristics. First, the leader of the course is a professional author, and the participants are presented to each other as readers and writers rather than by their diagnoses. This is meant to reduce participants' negative self-conception and self-stigmatisation and allow for other non-illness-dominated facets of their identity to be developed [21, 51, 105, 111, 117, 119]. Second, the co-conductor as co-participant allows participants to feel safe in the sense that they know that they themselves, and other participants, can get support if needed. Third, the course is given in a non-clinical community setting with a warm and welcoming, structured atmosphere, where the roles and rules of the activity are clear. Fourth, it is made explicit that the course is not about performing and already possessing writing skills, but about discussing literature and learning about writing forms. Fifth, it is explicitly stated that the narrative distance, i.e., the distance between the narrated self and the self who narrates, should be respected [38]⁠. The reflective discussion thus concerns the text, and not the person who wrote it. This means that the group format differs from the kind of therapy that explicitly addresses personal experience [76, 83, 90]. The protective distance allows participants to share experiences and feelings without disclosing if they are personal. This is meant to reduce anxiety and the extent to which other participants’ reflections are experienced as invading, thus promoting playfulness and spontaneity in the engagement with the writing tasks and reflective sessions. The playful attitude is meant to allow for participants to put words on difficult feelings and experiences that may reveal new facets of themselves. During reflective sessions, the creative attitude is expected to allow participants to practice mentalising, i.e., the motivation and ability to reflect on one’s own and others’ mental states, taking a plurality of perspectives into account, and integrating others’ perspectives in one’s own outlook. This may enhance the experience of self-efficacy, enhance connectedness between the participants, reduce self-stigmatisation and stimulate self-esteem and renegotiation of identity [24, 55, 57, 94, 107, 110].

#### Treatment as usual

Participants are recruited from OPUS (i.e., outreach treatment of young persons with psychosis symptoms) and F-ACT (Flexible Assertive Community Treatment). OPUS is a nationally implemented 2-year-long early intervention for people with first-episode psychosis. F-ACT is a community-based treatment model that provides flexible, multidisciplinary support to individuals with severe mental illness. OPUS and F-ACT both involve enriched and flexible assertive community treatment, psychoeducation including medication and side effects, relapse prevention and family involvement [40, 75, 109]. In OPUS, a multidisciplinary team consisting of a psychiatrist, a psychologist, a psychiatric nurse, an occupational therapist, and a social worker are assigned to each patient with a 1:12 caseload. In F-ACT, a multidisciplinary team consisting of a psychiatrist, a psychiatric nurse, and a social worker are assigned to each patient. For both OPUS and F-ACT, a primary staff member is in regular contact with the patient and responsible for coordinating the treatment elements. The treatment is individual and contingent upon patients’ needs [40, 75, 109].

#### Adherence to the intervention

Adherence is enhanced by focusing on the conductor-tandem collaboration and the co-conductor’s communication with the participants. This has been improved continuously during the pilot study. Attendance at each session will be recorded by the co-conductor.

### Outcomes and assessments

#### Baseline characteristics

Baseline characteristics of the participants are collected through self-report and register data; see Table 1. The baseline characteristics are collected to ensure that the active and control groups do not differ on any potentially biasing characteristics. For the pilot RCT the PSE will be administered for diagnostic validation.

**Table 1:** Participant characteristics

Variable	Source of collection
Age	Self-report
Sex	Self-report
Employment status	Self-report
Highest education	Self-report
Diagnoses	Register
Time since diagnosis	Register
Earlier use of mental health services	Register
Prior admissions (#)	Register
Antipsychotics	Register
Suicide attempt	Register
Admitted for alcohol and drug abuse	Register
Present state examination (pilot RCT)	Clinician rated

#### Outcome measures

In Table 2 the primary and secondary outcome measures are listed. The primary outcome measure is the Questionnaire about the process of recovery (QPR) which measures personal recovery as understood within the CHIME framework [74, 99, 114] measured at the end of the intervention, i.e. approximately 4.5 months after baseline. Secondary outcome measures include QPR six months after the end of the intervention (10.5 months after baseline), as well as other measures of personal recovery, mentalising, self-efficacy, functioning and quality of life measured at 4.5 months.

**Table 2:** Primary and secondary measures

**Outcome**		**Measure**	**Pre**	**Post (4.5 months)**	**Post (10.5 months)**	**Source of collection**
*Primary*						
	Overall personal recovery	QPR	x	x		SR
*Secondary*						
	*Personal recovery*					
	Overall personal recovery	QPR	x		x	SR
	Connectedness	TBS	x	x		SR
	Self-efficacy	SEPRS	x	x		SR
	Functioning	WSAS	x	x		SR
	Quality of life	MANSA	x	x		SR
	*Mentalising*					
	Overall mentalising	MentS	x	x		SR
	Alexithymia	TAS	x	x		SR

*SR* self-report questionnaire*; QPR* Questionnaire for the process of recovery; *TBS* Thwarted belongingness scale; *WSAS* Work and Social Adjustment Scale; *SEPRS* Self-efficacy for personal recovery scale; *MANSA* Manchester Short Assessment of Quality of life; *MentS* Mentalization scale; *TAS* Toronto Alexithymia Scale

The explorative outcome measures are shown in Table 3. These include the secondary measures at the 10.5 months follow-up in the full sample. The outcome measures for the active group at 4.5 months and 10.5 months, respectively, will be compared to the outcome measures for the control group at 4.5 months and 10.5 months, respectively (see Data analysis).

**Table 3:** Exploratory measures

Outcome	Measure	Sample	Pre	Post (4.5 months)	Post (10.5 months)	Source of collection
*Exploratory*						
Connectedness	TBS	full	x		x	SR
Self-efficacy	SEPRS	full	x		x	SR
Functioning	WSAS	full	x		x	SR
Quality of life	MANSA	full	x		x	SR
Overall mentalising	MentS	full	x		x	SR
Alexithymia	TAS	full	x		x	SR
Cognition	SCIP-D	subsample	x	x		T
Social cueing	TASIT 2A	subsample	x	x		T
Balancing of other-perspective	VISPT	subsample	x	x		T
Positive and negative symptoms	PANSS-6	subsample	x	x		CR
Functioning	GAF-F	subsample	x	x		CR

*SR* self-report questionnaire; *T* task; *CR* clincian-rated; *TBS* Thwarted belongingness scale; *WSAS* Work and Social Adjustment Scale; *SEPRS* Self-efficacy for personal recovery scale; *MANSA* Manchester Short Assessment of Quality of life; *MentS* Mentalization scale; *TAS* Toronto Alexithymia Scale; *SCIP-D* Screen for cognitive impairment in psychiatry; *TASIT 2A* The awareness of social inference test; *VISPT* Visual perspective-taking task; *PANSS-6* Positive and Negative Syndrome Scale; *GAF-F* Global Assessment of Functioning Scale

The additional explorative outcome measures of the pilot RCT are also shown in Table 3. These include measures of cognitive impairment, social cognition, balancing of other-perspective, psychosocial function, and symptom severity. The outcome measures will be compared between active and control group at 4.5 months, and between baseline and at 4.5 months to explore feasibility of the measures.

#### Safety measures

Safety measures are displayed in Table 4.

**Table 4:** Safety measures

Outcome	Measure	Pre	Post (4.5 months)	Post (10.5 months)	Source of collection
*Safety measures*	Admissions	x		x	register
	Deaths	x		x	register
	Suicide	x		x	register

### Assessment tools

#### Personal recovery

*Questionnaire for the process of recovery (QPR):* Personal recovery is measured using the Questionnaire for the process of recovery (QPR), a validated 15-item measure of personal recovery as conceptualised within the CHIME framework and developed in Britain as a collaboration between clinicians and mental health service users [53, 54, 74, 99, 114]. Each item is answered using a 5-point Likert scale with anchors “strongly disagree” and “strongly agree”, resulting in a range of 0–60. A mean difference of 4 has been considered relevant [53].

*Thwarted belongingness scale (TBS):* The recovery dimension of connectedness is measured using Thwarted belongingness scale (TBS), a validated 8-item measure of the subjective feeling of isolation and connectedness [39, 60]. Each item is answered on a 7-point Likert scale with anchors “not at all true for me” and “true for me”, resulting in a range of 8–56. A mean difference of 14 has been considered relevant [60].

#### Self-efficacy

*Self-efficacy for personal recovery scale (SEPRS):* Self-efficacy is measured with the Self-efficacy for personal recovery scale (SEPRS) [112], which comprises 14 items on the form “How confident are you that you can…”. Each item is answered on a scale from 1–100, resulting in a range of 14–1400.

#### Functioning

*The Work and Social Adjustment Scale (WSAS):* Impairment in social functioning is measured by the validated Work and Social Adjustment Scale (WSAS) [69]. WSAS is a self-report 5-item scale that assesses functioning on a scale from zero to eight. The sum score ranges between 0 and 40. A mean difference of 9 has been considered relevant [69]⁠.

#### Quality of life

*The Manchester Short Assessment of Quality of life (MANSA):* Quality of life is measured using the Manchester Short Assessment of Quality of life (MANSA). MANSA is a validated 16-item self-report questionnaire, developed primarily for persons with schizophrenia spectrum disorders and comprising items on whether the person i) has a friend ii) has a friend that s/he has seen in the last week, iii) has been accused of a crime, and iv) has been the victim of violence, as well as 12 items on satisfaction with occupation, activities, relationships, health, and living and financial situation [12, 84, 88]. The 12 items are answered on a 7-point Likert scale with anchors “couldn’t be worse” and “couldn’t be better”, resulting in a sum score in the range 12–84. A mean difference of 4 points has been considered relevant [11].

#### Mentalising

*Mentalization scale (MentS):* Mentalising is measured with the Mentalization scale, which is a validated 28-item scale consisting of three subscales Self-Related Mentalisation, Other-Related Mentalisation, and Motivation to Mentalise [3, 29]. It is scored on a 5-point Likert scale, resulting in a score range of 28–140. A mean difference of 8 has been considered relevant [29].

*Toronto Alexithymia Scale (TAS):* Alexithymia is measured using Toronto Alexithymia Scale (TAS), a validated 20-item scale with three subscales: difficulty describing feelings, difficulty identifying feelings, and externally oriented thinking, i.e., low motivation and habit to reflect on feelings [6, 7, 47, 66, 108]. Each item is answered on a 5-point Likert scale with anchors “not at all true for me” and “to a high degree true for me”, resulting in a range of 20–100. A mean difference of 7 has been considered relevant [66].

#### Additional assessment tools for the pilot RCT

*The Screen for cognitive impairment in psychiatry (SCIP):* The Screen for cognitive impairment in psychiatry (SCIP-D, Danish version) is used to measure cognitive performance [89]. The SCIP-D is a validated screening tool for detecting cognitive impairment in persons with psychotic and affective disorders [10, 80, 93]. A low score signifies more impairment. A mean difference of 3.6 has been considered relevant [97].

*Positive and Negative Syndrome Scale (PANSS-6):* The severity of positive and negative symptoms is measured by the Positive and Negative Syndrome Scale (PANSS-6), which is a 6-item clinician-rated scale measuring delusions, conceptual disorganisation, hallucination, blunted affect, social withdrawal, and lack of spontaneity in conversation. Each item is rated from 1–7, giving a possible score between 6 and 42 [48, 78, 79].

*Global Assessment of Functioning Scale – function (GAF-F):* Social function is measured using the Global Assessment of Functioning Scale – function [82, 86]. GAF-F is an interview-based, clinician-rated estimate of social function including occupational function, for which 4 points on a 100-point scale has been proposed a minimum clinically relevant effect ⁠[1, 23].

*The Awareness of Social Inference Test (TASIT):* Social cueing is measured with The Awareness of Social Inference Test (TASIT) [13]⁠, subsection 2A [44, 63]⁠. The TASIT assesses social cueing using videos of naturalistic everyday conversations in which two actors interact. Participants are asked questions about the communicative intentions of the people in the clips. The total accuracy score takes values in the range of 0–60. A mean difference in accuracy of 4 has been considered relevant [13]⁠.

*Visual perspective-taking task (VISPT):* Balancing of the other-perspective is measured with the Visual perspective-taking task (VISPT) [95]⁠. A computer-based task measuring ability or tendency to ignore another person’s perspective in one’s visual perception, and ability or tendency to take another person’s perspective into account in one’s visual perception judgments. Participants view an avatar in a room, with a number of discs on the walls: in one condition (consistent) the avatar sees the same number as the participant, in the other (inconsistent) the number differs. Correct scores range from 0–36 with correctness on consistent compared to inconsistent trial computed as a ratio [52, 100]⁠.

### Assignment of interventions

After baseline data are obtained, the participants are randomly allocated to either the control (standard mental healthcare) or active group (REWRITALIZE + standard mental healthcare) with a 1:1 allocation using the randomisation module in REDCap (Research Electronic Data Capture) [41, 42]. The randomisation sequence will be generated by an external researcher at Copenhagen Research Centre for Mental Health (CORE) and uploaded to REDCap. The randomisation is stratified by sites. Varying block sizes, unknown to the research team, are used. To ensure concealment, the randomisation schedule is stored away from the research team and the block sizes are not disclosed. Participants randomised to the control group will be set on a waiting list and will be informed that they will be offered to participate in a creative writing group within one year of the randomisation.

### Blinding

Owing to the nature of the intervention, neither participants, nor staff can be blinded to allocation. Participants will be instructed not to disclose the allocation status to the researchers. Baseline data will be collected prior to randomisation and those who collect follow-up data will be blinded. An employee outside the research team will extract data from REDCap on study completion, and group allocation will be coded with A and B to ensure blinding of the researchers during analysis and interpretation of data, drawing conclusions, and writing reports.

### Data collection and analysis

#### Data collection

Data will be collected at baseline and at follow-ups at 4.5 and at 10.5 months after baseline (see Table 5). After informed consent forms have been signed, participants can choose to fill out the questionnaires while the research assistant stays with them on Teams, or they can choose to do it after the meeting. Data collection for these active groups and their controls will commence approximately a month prior to group start and stop at the second follow-up at 10.5 months after group start. For each participant that drops out, the reason for drop-out will be registered. All participants who have not withdrawn their consent will be contacted for follow-ups, including those who decided to stop the intervention before it ended. For the pilot RCT data will be collected at baseline and follow-up at 4.5 months after baseline.

**Table 5:** Schedule of enrolment, interventions, and assessments

	**Study period**					
	**Enrolment**	**Allocation**	**Postallocation**			**Closeout**
**Time point**	t-1	t0	t1	t2	t3	t4
**Enrolment**						
Eligibility screen	x					
Informed consent	x					
**Allocation**		x				
**Interventions**						
REWRITALIZE			x			
TAU			x			
**Assessments**						
*Baseline characteristics*						
Age	x					
Sex	x					
Employment status	x					
Highest education	x					
Diagnoses	x					
Time since diagnosis	x					
Earlier use of mental health services	x					
Prior admissions (#)	x					
Antipsychotics	x					
Suicide attempt	x					
Alcohol and drug abuse	x					
*Outcome measures*						
Personal recovery QPR	x			x	x	
Connectedness TBS	x			x	x	
Self-efficacy SEPRS	x			x	x	
Functioning WSAS	x			x	x	
Quality of life MANSA	x			x	x	
Overall mentalising MentS	x			x	x	
Alexithymia TAS	x			x	x	
*Outcome measures (subset, exploratory)*						
Cognition SCIP-D	x			x		
Social cueing, TASIT 2A	x			x		
Balancing other-perspective VISPT	x			x		
Positive and negative symptoms PANSS-6	x			x		
Functioning GAF-F	x			x		
*Safety measures*						
Admissions	x					x
Deaths						x
Suicide attempt	x					x

*QPR* Questionnaire for the process of recovery; *TBS* Thwarted belongingness scale; *SEPRS* Self-efficacy for personal recovery scale; *WSAS* Work and Social Adjustment Scale; *MANSA* Manchester Short Assessment of Quality of life; *MentS* Mentalization scale; *TAS* Toronto Alexithymia Scale; *SCIP-D* Screen for cognitive impairment in psychiatry; *TASIT 2A* The awareness of social inference test; *VISPT* Visual perspective-taking task; *PANSS-6* Positive and Negative Syndrome Scale; *GAF-F* Global Assessment of Functioning Scale

Table 5 shows the participant timeline and an overview of the data collection in agreement with the standard protocol items for randomised clinical trials, recommendations for intervention trials (SPIRIT) [17]. Data are collected and stored using REDCap, an electronic data capture tool hosted at the Capital Region of Denmark. REDCap is a secure, web-based software platform designed to support data capture for research studies [41, 42].

#### Sample size and power calculations

The minimum sample size is calculated based on the ability to detect a minimal but clinically significant difference between the active intervention group and the control group in the primary personal recovery QPR measure. The minimal clinically significant difference between the study groups has been estimated to 4 points [53]. Based on trials in similar populations, we assume a standard deviation of 10 in the study population, which corresponds to an effect size of Cohen’s* d* = 0.4 [104, 114]. To achieve a statistical power of 90% at a significance level of 5%, a total of 266 participants must be included in this study to detect a difference: 133 in each group. Based on the needed participants, power calculations for secondary outcomes were calculated and are presented in Table 6. Because of the relatively high drop-out rate in the pilot study and similar studies [64, 110], we will plan for a sample size of 150 participants in the active group and 150 participants in the control group. This should hence allow for at least a 90% power of finding a minimally clinically significant difference in the primary measure, i.e., the QPR measure of personal recovery.

**Table 6:** Power

**Outcome**		**Measure**	**Alpha**	**Mean diff**	**SD**	**Cohen’s *d***	**Power**	***n***	**Mean diff source**	**SD source**
*Primary*			0,05							
	Personal recovery	QPR		4	10	0,40	0,9	266	[53]	[114]
*Secondary*										
	Connectedness	TBS	0,05	14	14	1	1	266	[60]	[60]
	Self-efficacy	SEPRS	0,05	x^a^	x	0,40	0,9	266		
	Functioning	WSAS	0,05	5	7	0,71	1	266	[69]	[69]
	Quality of life	MANSA	0,05	4	11	0,36	0,83	266	[11]	[11]
	Overall mentalising	MentS	0,05	8	12	0,66	1	266	[29]	[29]
	Alexithymia	TAS	0,05	7	11	0,63	1	266	[66]	[47]

See Table 5 and the assessment tool section for outcome measure abbreviations

^a^Power estimates for measures for which no relevant mean difference was identified are based on a moderate to small effect size of Cohen’s* d*=0.4

For the pilot RCT no calculation of sample size has been made as the primary aim for a pilot RCT is to explore design uncertainties before proceeding to a future RCT [35], here feasibility of chosen measures.

#### Data analyses

The main outcome measure is personal recovery measured by QPR. To test the research hypothesis, the differences at follow-up between the active intervention group and the control group will be analysed using independent-samples t-test⁠. Effect sizes to judge clinical relevance will be estimated by Cohen’s d [19]. All variables are continuous. Secondary and exploratory measures will be tested by the same means. The significance level is set to 0.05 for all measures. If there, despite randomisation, are differences in any baseline measure between active and control groups, these differences will be adjusted for in the comparison between active and control groups using general linear models.

Data analyses will be based on the intention-to-treat principle. Data from all participants will thus be included in the analyses. In accordance with the principle, missing data from the follow-up will be imputed using multiple imputations by means of Markov Chain Monte Carlo methods in Stata. All co-variates of supposed prognostic significance will be used to impute a distribution of missing data. A prerequisite for multiple imputation is that data are missing at random. This will be examined by various means, e.g. by comparing prognostic baseline characteristics between the participants for whom follow-up data are missing and the participants for whom follow-up data have been collected.

As a supplementary analysis, the differences at follow-up between the active and control groups will be analysed including only observed data, i.e. data for whom follow-up data have been collected. A detailed statistical analysis plan will be prepared and uploaded to clinicaltrials.gov before initiating analyses.

For the pilot study mean, standard deviation, within and between group differences on the outcome measures will be calculated.

### Monitoring

#### Data monitoring

Unexpected harms are collected during the study period through e-mail, telephone or face-to-face communication between participants and co-conductors. Unanticipated adverse events including drastic worsening of symptoms, aggressive behaviour or suicide in the writing group will be communicated by the co-conductor both to the clinicians at the psychiatric centre, and to the project group. The project group will discuss if measures need to be taken. Unexpected adverse events will be reported in trial publications.

#### Fidelity

A fidelity scale has been developed to ensure that the different centres and conductor-tandems (author + co-conductor pairs) comply with the REWRITALIZE design. The crucial features of the design that will be assessed include: i) The conductor-tandem is trained and supervised, and the author functions as leader and the co-conductor as one of the participants. ii) Participant are presented to each other as readers and writers rather than as persons with a diagnosis, and the sessions provide welcoming, calm, and structured spaces with snacks, coffee and providing of pens and paper. iii) It is emphasised that the discussion is about the text and not about the person, that playfulness, improvisation, and sharing and recognition is encouraged, and it is made clear that a multitude of perspectives is welcomed. iv) Participants are aware that the discussion is about the text and not about its author and that they do not have to reveal if a text is based on autobiographical material or not. Participants understand that it is about playfully trying out writing forms and discussing those and not about performing. v) Participants experience the space as safe and without focus on illness, and the co-conductor as someone they can talk to and trust. Participants experience the activity as meaningful. The program fidelity will be monitored by site approximately every six months.

## Discussion: perspectivation and limitations

The aim of this RCT is to assess if REWRITALIZE as add-on to standard mental healthcare is more effective than standard mental healthcare alone for promoting personal recovery. This is the first RCT for creative writing groups and participatory arts. The study design closely follows the SPIRIT guidelines. With the planned sample size, it has at least 90% power of finding a medium to small effect size for the primary measure of personal recovery. If we can show that there is a difference between the active group and the control group, this would indicate that participation in REWRITALIZE increases participants’ chance of recovery. As this would constitute evidence for the efficacy of REWRITALIZE, it would allow the intervention to be offered by mental health centres across Denmark on evidence-based grounds. Since this is a multicentre study, this aspect of the study design may facilitate the implementation at mental health centres.

The kind of control group – the waiting list control – was chosen because this is the first RCT conducted for this intervention. To enable testing of the beneficial effect of the artistic activity per se, the control group should take part in another non-artistic social activity preferably involving non-artistic writing.

One first limitation of the current RCT is that the use of mental health services is not recorded. It may thus be that the use of mental health services differs between the active and control groups. Second, participants were not asked to refrain from participation in other participatory arts groups. It is hence possible that those allocated to the control group took part in other participatory arts groups outside of mental health centres. Third, since the candidate participants did not see a clinician prior to enrolment, no exclusion criteria could be implemented. Some patients may be excluded by the co-conductor after enrolment because they are judged to display antisocial behaviour or use alcohol and drugs in a way that hinders participation in the intervention. Lastly, the nature of the intervention was such that participants could not themselves be blinded to which group they were in.

Furthermore, the embedded pilot study is the first to address if the chosen measures are feasible for assessing potential effects of creative writing on social cognition and cognition in a clinical and controlled setting. Analyses of the pilot RCT will provide important knowledge regarding the available measures and inform the design of a full-scale trial investigating the possible effectiveness of a creative writing group intervention on cognitive, social cognitive and psychosocial functioning, and symptom reduction.

## Data Availability

No datasets were generated or analysed during the current study.

## Abbreviations

CHIME: Connectedness, Hope, Identity, Meaning, Empowerment
CORE: Copenhagen Research Centre for Mental Health
F-ACT: Flexible Assertive Community Treatment
GAF-F: Global Assessment of Functioning Scale-Function
MANSA: Manchester Short Assessment of Quality of Life
MentS: Mentalization Scale
NHS: National Health Service
PANSS: Positive and Negative Syndrome Scale
PSE: Present State Examination
QPR: Questionnaire for the Process of Personal Recovery
REDcap: Research Electronic Data Capture
RCT: Randomised Controlled Trial
SCIP: Screen for Cognitive Impairment in Psychiatry
SEPRS: Self-Efficacy for Personal Recovery Scale
SPIRIT: Standard Protocol Items: Recommendations for Interventional Trials
TAS: Toronto Alexithymia Scale
TASIT: The Awareness of Social Inference Test
TBS: Thwarted Belongingness Scale
VISPT: Visual Perspective-Taking task
WSAS: Work and Social Adjustment Scale
